# Annexin A5 suppresses cyclooxygenase-2 expression by downregulating the protein kinase C-ζ–nuclear factor-κB signaling pathway in prostate cancer cells

**DOI:** 10.18632/oncotarget.19392

**Published:** 2017-07-19

**Authors:** Hyoung-Seok Baek, Nahee Park, Yeo-Jung Kwon, Dong-Jin Ye, Sangyun Shin, Young-Jin Chun

**Affiliations:** ^1^ College of Pharmacy and Center for Metareceptome Research, Chung-Ang University, Seoul 06974, Republic of Korea

**Keywords:** ANXA5, COX-2, p65, PKC-ζ, auranofin

## Abstract

Annexin A5 (ANXA5) is a member of the annexin protein family. Previous studies have shown that ANXA5 is involved in anti-inflammation and cell death. However, the detailed mechanism of the role of ANXA5 in cancer cells is not well understood. In this study, we investigated the inhibitory effect of ANXA5 on cyclooxygenase-2 (COX-2) in prostate cancer cells. Expression of COX-2 induced by TNF-α was inhibited by overexpression of ANXA5 and inhibition of COX-2 expression by auranofin, which could induce ANXA5 expression, was restored by ANXA5 knockdown. In addition, ANXA5 knockdown induces phosphorylation of NF-κB p65 in prostate cancer cells, indicating that ANXA5 causes COX-2 downregulation through inhibition of p65 activation. We also found that protein kinase C (PKC)-ζ protein levels were upregulated by the inhibition of ANXA5, although the mRNA levels were unaffected. We have shown that upregulated COX-2 expression by inhibition of ANXA5 is attenuated by PKC-ζ siRNA. In summary, this study demonstrates that downregulation of PKC-ζ-NF-κB signaling by ANXA5 may inhibit COX-2 expression in prostate cancer.

## INTRODUCTION

There is increasing evidence that annexin A5 (ANXA5) has roles in cytotoxicity [1, 2], apoptosis [3, 4], and anti-inflammatory effects [5]. However, the detailed molecular mechanisms of ANXA5 in cancer cells are not yet fully understood. Previously, we found that auranofin induces expression of ANXA5 in human prostate cancer cells and triggers apoptosis [3]. Auranofin is a well-known lipophilic gold compound that has anti-inflammatory effects and immunosuppressive properties. Consequently, auranofin has been widely used for the treatment of rheumatoid arthritis (RA). A number of studies showed that auranofin inhibits production of proinflammatory cytokines, such as tumor necrosis factor alpha (TNF-α), interleukin-1β (IL-1β), interleukin-6 (IL-6), and cyclooxygenase-2 (COX-2) expression [6-8]. In particular, the repressive effect of COX-2 has been previously emphasized [9, 10].

Several studies suggest a correlation between inflammation and prostate cancer. Nelson et al [11] and Dennis et al [12] showed that inflammation is a risk factor for development of prostate cancer. Moreover, Wang et al [13] has shown that COX-2 expression affects carcinogenic process and chemopreventive effects of anti-inflammatory drugs in prostate cancer cells. Therefore, it is important to demonstrate the detailed mechanism of COX-2 inhibitory effect by ANXA5 in prostate cancer cells for the improvement of prostate cancer chemotherapy.

Phosphorylation and activation of the p65 subunit of NF-κB have an essential role in the regulation of inflammatory cytokines [14-16]. Several researchers have shown that NF-κB activation induces the increase of COX-2 expression and inflammatory response both *in vitro* and *in vivo* by intra and extracellular stimuli [17-19]. The Ser536 residue of p65 is important for p65 translocation into the nucleus [20], being involved in IκB kinase (IKK)-mediated IκBα phosphorylation and subsequent ubiquitin-dependent degradation [21]. IKK activity in primary fibroblast-like synoviocytes was significantly increased within 10 min of stimulation by TNF-α and IL-1, accompanied by phosphorylation and degradation of IκBα [22]. Phosphorylation of p65 is not only related to activation of p65 nuclear translocation, but to induction of p65 transactivation activity. Phosphorylation of Ser311 has been shown to be important for interaction of p65 with the cAMP response element-binding protein (CBP)/p300 [23]. The interaction between p65 and CBP induces p65 transactivation and then promotes expression of inflammatory cytokines [24].

Previous studies have shown that PKC-ζ acts as a signaling component upstream of transforming growth factor β-activated kinase 1 (TAK1) and downstream of RhoA and induces activation of NF-κB in macrophages by lipopolysaccharide (LPS) [25]. PKC-ζ is one of the well-known kinase, which phosphorylates Ser536 and Ser311 residues of p65 to promote nuclear translocation and transactivation [24, 26].

ANXA5 has been shown to interact with some PKCs. PKC-α is directly coupled with ANXA5 to cause apoptotic events and PKC-δ translocation is upregulated through a direct interaction between ANXA5 and PKC-δ which participate in cellular signaling events [27-29], but interaction between ANXA5 and PKC-ζ has not yet been confirmed. In this study, we determined that inhibition of ANXA5 induces COX-2 expression in human prostate cancer cells and explored the correlation between PKC-ζ and ANXA5 on the regulation of COX-2 expression. Our results suggest that ANXA5 may act as a negative regulator for COX-2 expression by downregulating the PKC-ζ-NF-κB pathway in prostate cancer cells.

## RESULTS

### Auranofin inhibits COX-2 expression in PC-3 cells

**Figure 1:**
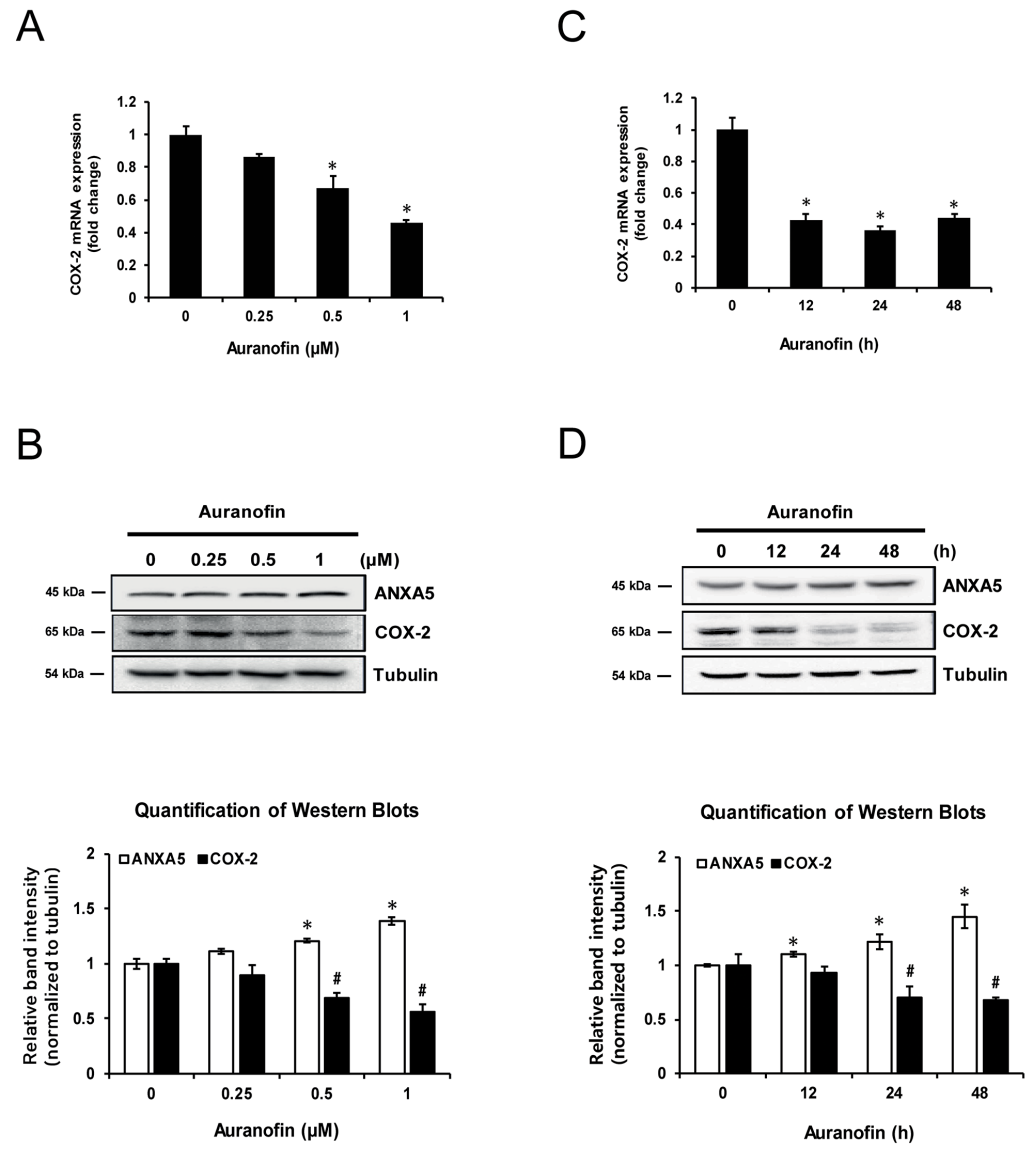
Inhibition of cyclooxygenase 2 (COX-2) expression by auranofin in PC-3 cells **(A-B)** Cells were treated with the indicated concentration of auranofin for 24 h. (A) Expression of COX-2 mRNA was determined using qPCR. (B) COX-2 proteins were measured using western blot analysis. Quantification of western blots (n = 3). **(C-D)** Cells were treated with 1 μM auranofin for 12, 24, or 48 h. (C) Expression of COX-2 mRNA was determined using real-time qPCR. (D) COX-2 proteins were measured using western blot analysis. Quantification of western blots (n = 3). *^,^^#^P < 0.05.

Previously, we showed that auranofin induces ANXA5 in human prostate cancer PC-3 cells [3]. Auranofin is already known to inhibit COX-2 expression in synovial cells [10]. PC-3 cells were treated with auranofin in order to determine whether it is also able to inhibit COX-2 expression in PC-3 cells. After treatment with auranofin (0.25, 0.5, or 1 μM) for 24 h, COX-2 levels were measured by quantitative PCR (qPCR) and western blot (Figure 1A and 1B). Auranofin significantly inhibited COX-2 mRNA and protein expression, and this occurred in a time-dependent manner (Figure 1C and 1D). These data showed that auranofin suppresses COX-2 expression in PC-3 cells in concentration- and time-dependent manners.

### Inhibition of ANXA5 induces COX-2 expression in PC-3 cells

**Figure 2:**
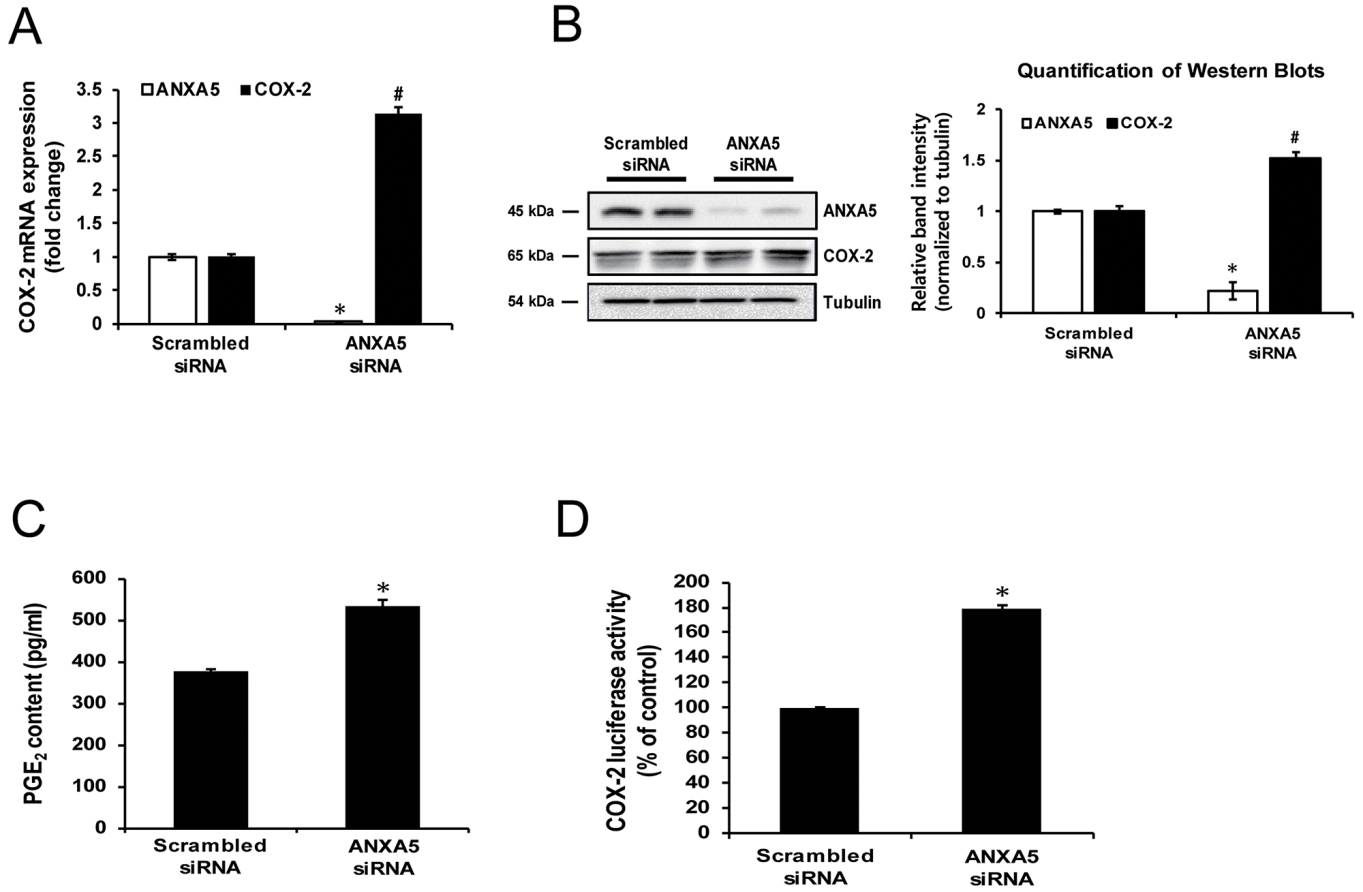
Effect of annexin A5 knockdown by siRNA on cyclooxygenase 2 (COX-2) expression **(A-D)** PC-3 cells were transfected with scrambled or annexin A5 siRNA for 48 h. (A) Expression of COX-2 mRNA was determined using qPCR. (B) COX-2 proteins were measured using western blot analysis. Quantification of western blots (n = 3). (C) PGE2concentration in cell supernatants was measured by ELISA. (D) COX-2 promoter activity was determined using dual luciferase assay. *, ^#^P < 0.05.

To determine whether ANXA5 exhibits an inhibitory effect on COX-2 expression, PC-3 cells were transfected with ANXA5-specific siRNA. Knockdown of ANXA5 increased COX-2 mRNA and protein levels by 3-fold and 1.5-fold, respectively (Figure 2A and 2B). However, in other cancer cell lines, including human hepatoma cells Hep3B and HuH-7, human cervical cancer cells HeLa, and human breast cancer cells MCF-7 and MDA-MB-231, ANXA5 knockdown showed no significant effects on COX-2 expression (Supplementary Figure 1). Prostaglandin E2 (PGE2) is a well-known metabolite produced by COX-2. A number of studies confirmed the alteration of PGE2 content in association with COX-2 expression [30-33]. Thus, to demonstrate whether the change in COX-2 expression induced by ANXA5 siRNA influenced prostaglandin metabolism, alteration in PGE2 production in cell culture media was measured. As shown in Figure 2C, PGE2 production was increased 1.4-fold by ANXA5 siRNA. Additionally, to determine whether ANXA5 controlled COX-2 promoter activity, a dual-luciferase reporter assay was performed (Figure 2D). Similar to previous data, COX-2 promoter activity was increased by more than 80% compared to the control when cells were treated with ANXA5 siRNA (37.5 nM) for 48 h.

### Induction of ANXA5 by auranofin inhibits COX-2 expression in prostate cancer cells

**Figure 3:**
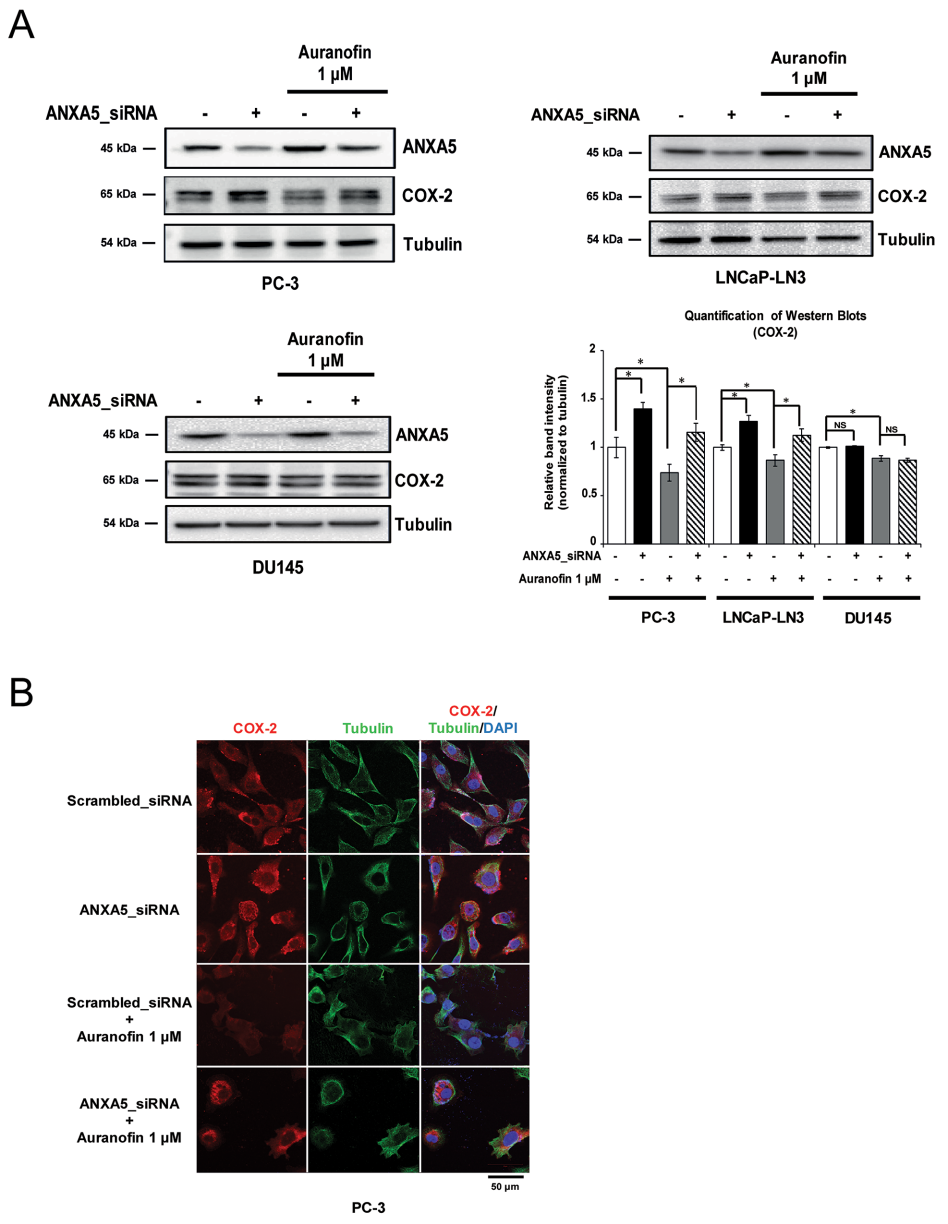
Rescue of cyclooxygenase 2 (COX-2) expression in auranofin-treated prostate cancer cells by inhibition of annexin A5 **(A-B)** Cells were transfected with annexin A5 siRNA and then treated with auranofin (1 μM) for 24 h. (A) Western blot analysis was performed using whole cell lysates of PC-3, LNCaP-LN3, and DU145 cells. Quantification of western blots (n = 3). (B) Confocal analysis was performed to detect COX-2 expression. Microscopy scale bar = 50 μm. NS = not significant, *P < 0.05.

We next determined whether the inhibition of COX-2 expression by auranofin was rescued by ANXA5 knockdown in prostate cancer cells. PC-3, LNCaP-LN3, and DU145 cells were transfected with ANXA5 siRNA (37.5 nM) for 48 h in the presence of auranofin (1 μM) (Figure 3A). Western blot analysis revealed that COX-2 expression was rescued in ANXA5 knockdown PC-3 and LNCaP-LN3 cells compared to auranofin-only-treated cells. The data from confocal microscopy analysis also showed that COX-2 levels decreased by auranofin were recovered by ANXA5 knockdown (Figure 3B). In contrast with the results in PC-3 and LNCaP-LN3 cells, ANXA5 siRNA did not show any significant effect in DU145 cells. Taken together, our data demonstrate that auranofin-mediated induction of ANXA5 may play a crucial role in the inhibition of COX-2 expression in PC-3 and LNCaP-LN3 cells.

### Overexpression of ANXA5 represses TNF-α-mediated COX-2 induction

**Figure 4:**
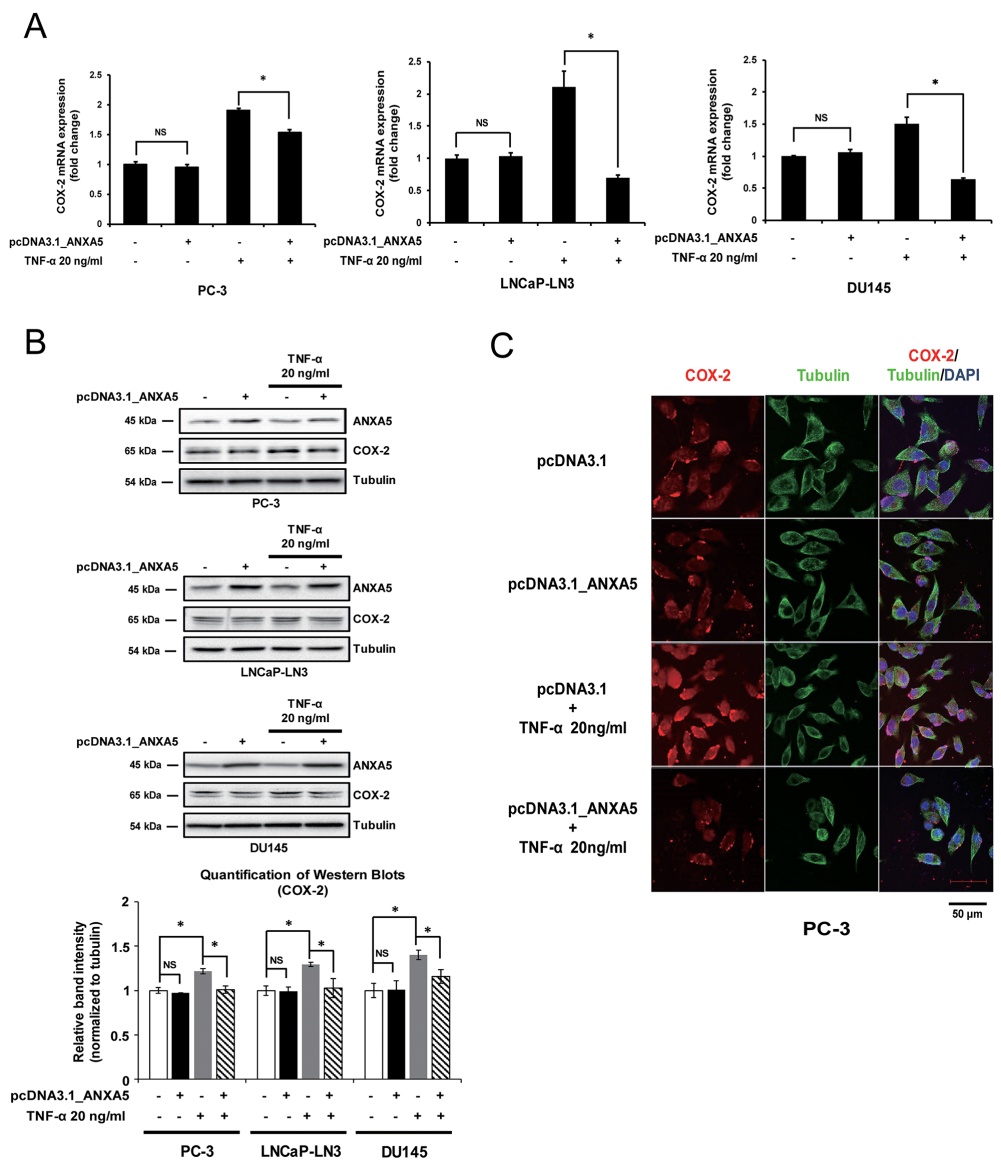
Suppression of tumor necrosis factor α (TNF-α)-induced cyclooxygenase 2 (COX-2) expression by overexpression of annexin A5 **(A-B)** PC-3, LNCaP-LN3, and DU145 cells were transfected with annexin A5 expression vector and then treated with TNF-α (20 ng/μL) for 4, 6, and 1 h, respectively. (A) qPCR was performed to detect the expression of COX-2 mRNA. (B) Western blot analysis was performed by using whole cell lysates. Quantification of western blots (n = 3). **(C)** Confocal analysis was performed to detect COX-2 expression. Microscopy scale bar = 50 μm. NS = not significant, *P < 0.05.

Next, we assessed the contribution of ANXA5 to the regulation of COX-2 expression by overexpressing ANXA5. Although there was no significant change in COX-2 mRNA and protein levels (Figure 4A and 4B), ANXA5 overexpression inhibited COX-2 expression when cells were treated with TNF-α (20 ng/mL), which is known to induce COX-2 mRNA expression in various prostate cancer cells, including PC-3, LNCaP-LN3, and DU145 [34]. COX-2 protein levels were also decreased by ANXA5 overexpression in TNF-α-stimulated cells (Figure 4B). Confocal microscopy analysis confirmed that ANXA5 overexpression suppressed TNF-α-induced COX-2 expression (Figure 4C). These results indicate that ANXA5 may downregulate TNF-α-induced COX-2 expression in prostate cancer cells.

### ANXA5 may block NF-κB p65 activation

**Figure 5:**
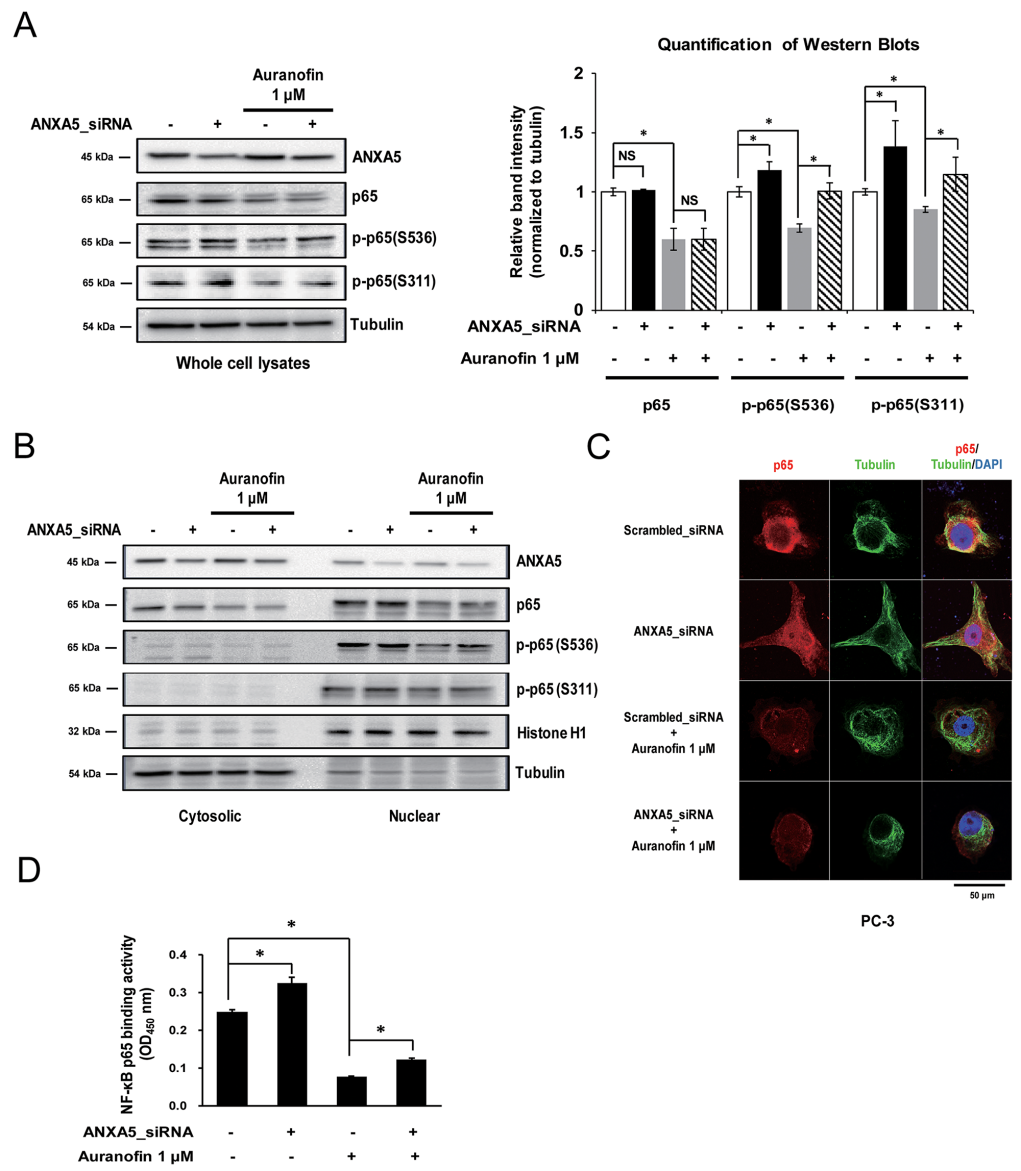
Phosphorylation of the nuclear factor-κB (NF-κB) p65 subunit by inhibition of annexin A5 **(A-D)** PC-3 cells were transfected with annexin A5 siRNA and then treated with auranofin (1 μM) for 24 h. (A) Whole cell lysates were subjected to western blot analysis for p65, p-p65 (Ser536), and p-p65 (Ser311). Quantification of western blots (n = 3). (B) Nuclear and cytosolic lysates were subjected to western blot analysis for p65, p-p65 (Ser536), and p-p65 (Ser311). (C) Binding activity assay of the NF-κB p65 subunit was performed by using the nuclear lysates. (D) Confocal analysis was performed to detect p65 expression and location. Microscopy scale bar = 50 μm. NS = not significant, *P < 0.05.

NF-κB is one of the most well-known proinflammatory transcription factors. Therefore, to clarify the molecular mechanism of ANXA5 downregulation of COX-2 expression, we first identified the alteration of NF-κB p65 subunit level using whole cell lysates. Previous studies have shown that auranofin reduced NF-κB p65 expression in RAW 264.7 cells [35]. As shown in Figure 5A, our data also confirmed that auranofin (1 μM) inhibits p65 expression in PC-3 cells. We found that treatment with ANXA5 siRNA did not change the total p65 level. However, phosphorylation of p65 on Ser536 and Ser311 was increased by ANXA5 knockdown, and auranofin-mediated suppression of p65 phosphorylation was rescued by ANXA5 siRNA (Figure 5A). According to previous studies, these phosphorylations of p65 increase the translocation of p65 into the nucleus and induce the transcription of proinflammatory genes such as *IL-6*, *TNF-α*, and *COX-2* [20, 24]. To identify whether p65 translocation was increased by inhibition of ANXA5, we compared the p65 levels in cytosolic and nuclear fractions (Figure 5B). ANXA5 siRNA increased the p65 level and phosphorylation of p65 on Ser536 in the nucleus, whereas the cytosolic p65 level was decreased. Confocal microscopic analysis also showed nuclear translocation of p65 in PC-3 cells (Figure 5C). To investigate whether p65 phosphorylation by ANXA5 knockdown leads to activation of p65 transcriptional activity, NF-κB p65 binding activity was measured after transfection with ANXA5 siRNA in auranofin-treated cells (Figure 5D). Auranofin significantly inhibited the binding activity of p65; however, ANXA5 knockdown recovered the p65 binding activity decreased by auranofin. These results suggested that inhibition of ANXA5 induces p65 phosphorylation and subsequently activates p65 transcriptional activity in PC-3 cells.

### Inhibition of ANXA5 affects COX-2 expression by increasing the PKC-ζ protein level

**Figure 6:**
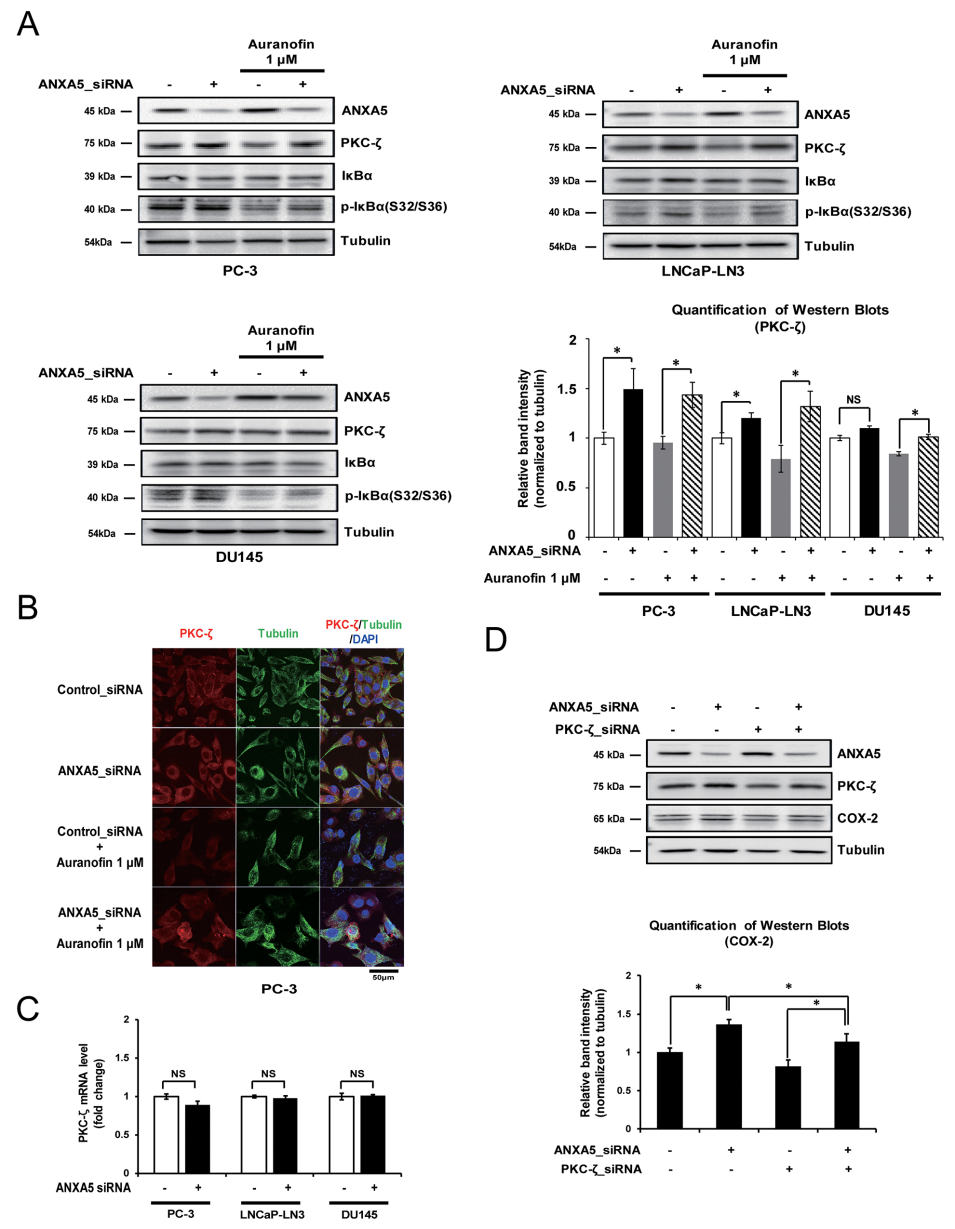
Effect of annexin A5 on protein kinase c (PKC)-ζ expression **(A-B)** Cells were transfected with annexin A5 siRNA and then treated with auranofin (1 μM) for 24 h. (A) Western blot analysis was performed to detect PKC-ζ expression by using PC-3, LNCaP-LN3, and DU145 whole cell lysates. Quantification of western blots (n = 3). (B) Confocal analysis was performed to detect PKC-ζ expression. **(C)** PC-3, LNCaP-LN3, and DU145 cells were transfected with annexin A5 siRNA, and qPCR was performed to detect the expression of PKC-ζ mRNA. **(D)** PC-3 cells were cotransfected with annexin A5 and PKC-ζ siRNA, and western blot analysis was performed to detect COX-2 expression by using the whole cell lysate. Microscopy scale bar = 50 μm. NS = not significant, *P < 0.05.

It has been reported that phosphorylation of p65 on Ser536 and Ser311 is affected by PKC-ζ [24, 26]. Therefore, to identify whether the phosphorylation of p65 after ANXA5 knockdown occurs via the induction of PKC-ζ expression, we first examined the alteration of PKC-ζ protein levels under the same conditions shown in Figure 5A. PKC-ζ protein levels were increased by ANXA5-knockdown in PC-3 and LNCaP-LN3 cells (Figure 6A). Confocal microscopic analysis showed that ANXA5 siRNA recovered the reduced PKC-ζ expression seen in auranofin-treated cells (Figure 6B). However, in DU145 cells, PKC-ζ expression was not affected by ANXA5 knockdown, although, when auranofin-treated DU145 cells were transfected with ANXA5 siRNA, the PKC-ζ protein level was slightly increased (Figure 6A). Because phosphorylation and degradation of IκBα may induce phosphorylation of p65 at Ser536 [22], it might be similarly induced by ANXA5 siRNA if PKC-ζ promotes phosphorylation of IκBα. As expected, increased phosphorylation of IκBα was shown by ANXA5 knockdown (Figure 6A). Interestingly, although PKC-ζ protein levels were increased by ANXA5 knockdown, PKC-ζ mRNA levels were not affected, indicating that ANXA5 may control PKC-ζ expression at the post-translational level (Figure 6C). To understand whether induction of COX-2 expression by ANXA5 siRNA is promoted via increased PKC-ζ expression, PC-3 cells were transfected with ANXA5 and PKC-ζ siRNA. PKC-ζ siRNA repressed the induction of COX-2 expression triggered by ANXA5 knockdown (Figure 6D). Therefore, these results suggest that inhibition of ANXA5 promotes COX-2 expression via induction of increased PKC-ζ protein levels.

## DISCUSSION

ANXA5 is widely distributed and abundantly expressed in cancer cells. In previous studies, ANXA5 stimulated various cellular responses, such as apoptosis and anti-inflammation [2, 3, 5]. However, the detailed role of ANXA5 in anti-inflammation and the signaling pathways involved have not been fully elucidated. Here we demonstrate that ANXA5 knockdown induces COX-2 expression, mediated by phosphorylation of p65 and ANXA5 overexpression inhibits TNF-α-induced COX-2 expression. Previous study showed that TNF-α induces phosphorylation of IκBα on Ser32 and Ser36 and induces p65 phosphorylation on Ser536 [36]. Therefore, ANXA5 overexpression may suppress TNF-α-induced COX-2 expression through interfering the IκBα phosphorylation and degradation.

Arnold *et al.* [5] showed that recombinant human ANXA5 inhibits LPS binding to thetoll-like receptor 4 (TLR4)/MD-2 receptor complex, leading to reduced Akt and NF-κB signaling. However, the detailed mechanism remained unclear. In our study, we showed that ANXA5 knockdown induces phosphorylation of the p65 subunit. Although there are several phosphorylation sites in p65, we confirmed that phosphorylation of p65 at Ser536 and Ser311 may play an important role in COX-2 expression [24, 37]. In particular, the phosphorylation of p65 at Ser536 is associated with translocation into the nucleus [20]. Confocal microscopic analysis and western blot data revealed that the nuclear p65 level is increased by ANXA5 knockdown.

PKC-ζ is able to induce phosphorylation of p65 at both Ser536 and Ser311 [24, 26] and increases the induction of COX-2 expression in response to proinflammatory stimuli [38]. In the present study, we found that ANXA5 knockdown increases PKC-ζ protein levels, and suppression of PKC-ζ by siRNA decreases the induction of COX-2 expression caused by ANXA5 knockdown. Thus, we believe that ANXA5 may play protective roles in the NF-κB-PKC-ζ pathway, causing suppression of COX-2.

Unlike other prostate cancer cells, no noticeable alteration of PKC-ζ expression level by ANXA5 knockdown was observed in DU145 cells, which is similar to the pattern of COX-2 expression. Previous studies show that differences in the basal expression level of specific genes in cells obtained from the same organ indicate different responses to extracellular stimuli [39-41]. Shabbeer et al [42] showed that differences in the basal expression levels of proliferating cell nuclear antigen between PC-3 and DU145 cells affect relatively high resistance of PC-3 cells to valproic acid as compared to DU145 cells. We found that DU145 cells expressed higher levels of PKC-ζ than PC-3 and LNCaP-LN3 cells (Supplementary Figure 2). Accordingly, we hypothesized that the increased expression of PKC-ζ in DU145 explains their weak response to ANXA5 knockdown-derived COX-2 and PKC-ζ expression. In addition, PKC-ζ mRNA levels were not affected by ANXA5 knockdown in PC-3, LNCaP-LN3, or DU145 cells.

In a recent study, Xin *et al.* [43] showed that mammalian target of rapamycin complex (mTORC)-2 phosphorylates the PKC-ζ turn motif, which protects against proteasome-mediated degradation and induces kinase activity. Notably, these effects are associated with phosphorylation at Thr560, a site known to be regulated by autophosphorylation [44]. Chou *et al.* [45] showed that phosphoinositide-dependent kinase-1 (PDK-1) activates phosphorylation of PKC-ζ at the activation loop (Thr410) and subsequently induces PKC-ζ autophosphorylation at Thr560 and PKC-ζ activity. We also found increased phosphorylation of PKC-ζ at Thr410 after ANXA5 knockdown (data not shown). Thus, we suggest that ANXA5 may inhibit phosphorylation of the PKC-ζ activation loop, subsequently interfering with PKC-ζ autophosphorylation at Thr560 to cause PKC-ζ degradation and reduce NF-κB signaling. The possibility that ANXA5 directly interacts with PKC-ζ remains to be elucidated.

Unlike PDK-1-dependent activation, Standaert *et al.* [46] showed that phosphatidylinositol 3,4,5 trisphosphate (PIP3) activates PKC-ζ autophosphorylation at Thr560 independent of phosphorylation of the activation loop. In addition, a previous report showed that p38 kinase also regulates PKC-ζ autophosphorylation at Thr560 [47]. Activated p38 can interact with the PKC-ζ regulatory domain and inhibits autophosphorylation. Although the mechanism of regulation of PKC-ζ translation and/or stability by ANXA5 has not been completely demonstrated, our data suggest that ANXA5 may regulate several kinases, including p38, which interact with PKC-ζ phosphorylation sites. Thus, future studies will be needed to understand the control mechanisms of PKC-ζ protein levels by ANXA5 without transcriptional changes, and elucidate whether differences in reactivity in response to proinflammatory stimuli are affected by the basal level of PKC-ζ expression in other cancer cells.

**Figure 7:**
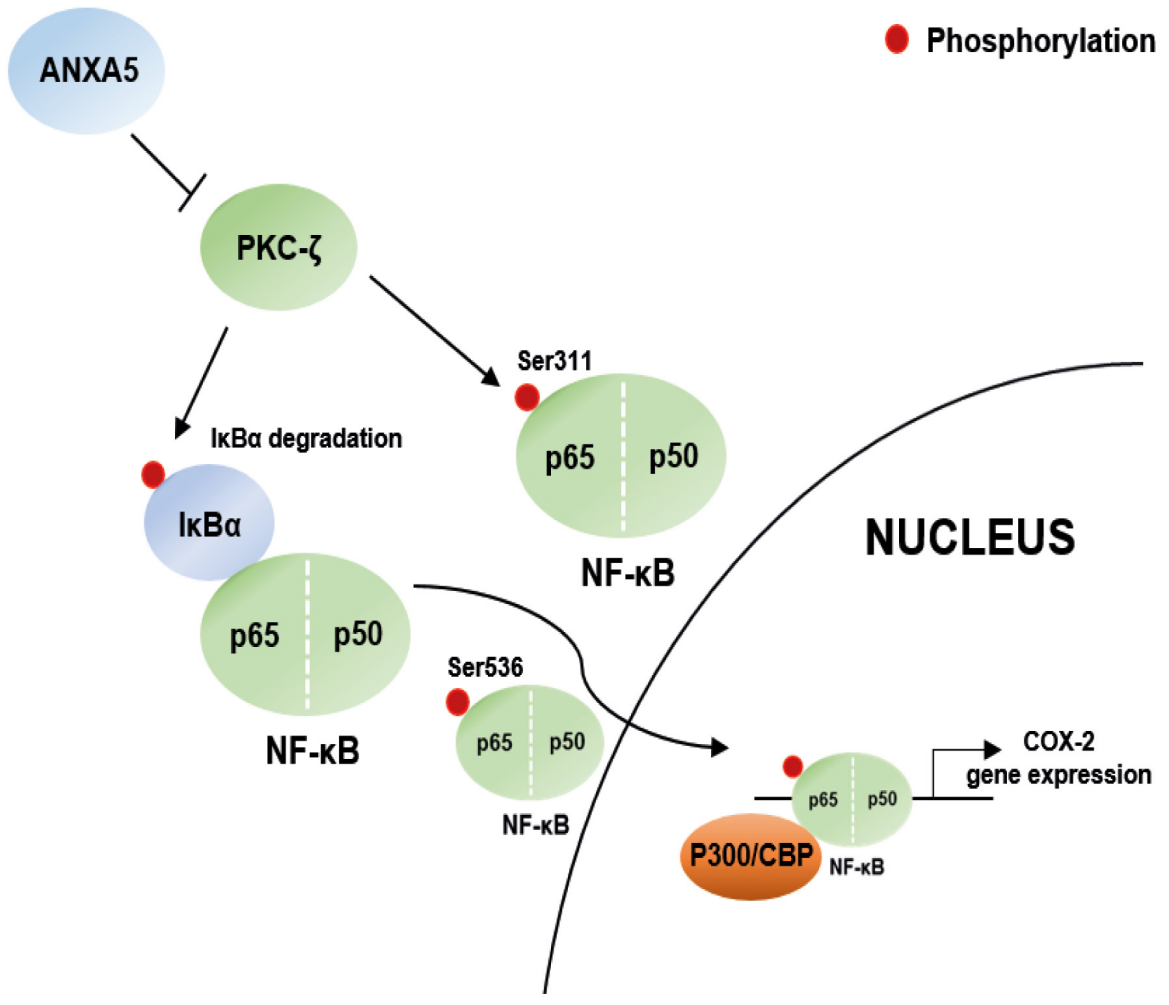
Scheme for the novel mechanisms of ANXA5 Scheme for ANXA5 suppressed COX-2 expression via PKC-ζ – NF-κB signaling in prostate cancer cells.

In summary, we demonstrated that ANXA5 suppresses COX-2 expression via inhibition of NF-κB p65 phosphorylation, which is regulated by alteration of PKC-ζ protein levels in prostate cancer cells. The scheme in Figure 7 summarizes these signaling pathway. Our results indicate that ANXA5 may have a crucial protective role in the cellular inflammatory response to proinflammatory stimuli. Therefore, we anticipate that applying the anti-inflammatory effect of ANXA5 to prostate cancer therapy will have a better effect on inhibition of cancer development and improvement of drug resistance.

## MATERIALS AND METHODS

### Cell culture

Human prostate cancer cell lines PC3, DU145, and LNCaP-LN3, and the human cervical cancer cell line HeLa were obtained from the Korean Cell Line Bank (Seoul, Korea). Human hepatocellular carcinoma cell lines Hep3B and HuH-7, and human breast cancer cell lines MDA-MB-231 and MCF-7 were obtained from the American Type Culture Collection (Manassas, VA). All cells were cultured in RPMI 1640 supplemented with 10% (v/v) heat-inactivated fetal bovine serum (FBS), 100 U/mL penicillin, and 100 μg/mL streptomycin. Cells were maintained at 37 °C in a humidified atmosphere of 5% CO_2_.

### Reagents

Auranofin was purchased from Sigma-Aldrich (St. Louis, MO). A solution of auranofin was prepared in dimethyl sulfoxide, stored as small aliquots at -20 °C, and then diluted as needed in cell culture medium. FBS and RPMI 1640 medium were purchased from HyClone (Logan, UT). The Neon transfection system was from Life Technologies (Carlsbad, CA). The bicinchoninic acid (BCA) protein assay kit and ECL kit were from Thermo Scientific (Waltham, MA). PGE2 ELISA assay kit was purchased from Enzo Life Science (Farmingdale, NY). Antibodies against tubulin (sc-8035) and goat anti-rabbit IgG-Texas Red (sc-2780), and Ultra Cruz™ mounting medium (sc-24941) were from Santa Cruz Biotechnology (Santa Cruz, CA). COX-2 antibody (A303-600A-M) was from Bethyl (Montgomery, TX). NF-κB (p65) (CSB-PA003436), p-p65 (S536) (CSB-PA000586), p-p65 (S311) (CSB-PA000719), and PKC-ζ (CSB-PA003805) antibodies were from Flarebio (College Park, MD). All other chemicals were of the highest purity or molecular biology grade and were obtained from commercial sources.

### Transient transfection

ANXA5 SMART pool siRNA (Dharmacon, Lafayette, CO) and the overexpression vector pcDNA3.1/Zeo^+^ containing the ANXA5-encoding sequence were used for transfection. Cells were transfected with 37.5 nM siRNA or 5 μg plasmid with the Neon Transfection System (Life Technologies) and cultured in 60-mm dishes in antibiotic-free RPMI with 10% FBS for 48 h.

### Quantitative PCR

Total RNA was extracted using a Ribospin™ kit (GeneALL, Seoul, Korea). Total RNA (0.5 μg) was reverse-transcribed at 37 °C for 1 h in 20 μL total volume containing 5× RT buffer, 10 mM dNTPs, 40 U RNase inhibitor, 200 U Moloney murine leukemia virus reverse transcriptase, and 100 pmol oligo-dT primer. qPCR was performed using the Rotor-Gene SYBR1 PCR Kit, as recommended by the manufacturer, and analyzed by using QIAGEN Rotor-Gene Q Series software. Each reaction contained 10 μL of 2×SYBR1 Green PCR Master Mix, 1 μM oligonucleotide primers, and 2 μL of cDNA in a final volume of 20 μL. Amplification was conducted as follows: one cycle at 95 °C for 5 min, followed by 40 cycles of denaturation at 95 °C for 5 s and annealing/extension at 56 °C for 10 s. The following primer sets were used for qPCR: COX-2, 5′-CTTGGGCACAGAGAGCA-3′ and 5′-AACTGCTCATCACCCCATTC-3′; 18S rRNA, 5′-GTAACCCGTTGAACCCCATT-3′ and 5′-CCATCCAATCGGTAGTAGCG-3′; PKC-ζ, 5′-AGAGCCTCCAGTAGACGACAA-3′ and 5′-CGGGATGAGGAAATGTAAGCAA-3′.

### Western blot analysis

Cells were solubilized with ice-cold lysis buffer containing 50 mM Tris-HCl (pH 7.4), 1% NP-40, 150 mM NaCl, 0.5% Na-deoxycholate, 2 mM EDTA, 0.1% SDS, and 50 mM NaF. The extracted proteins (20 μg) were separated by sodium dodecyl sulfate-polyacrylamide gel electrophoresis (SDS-PAGE) on 10–12% polyacrylamide gels, and electrophoretically transferred onto 0.45 μm PVDF membranes. Membranes were blocked with 5% (w/v) bovine serum albumin in Tris-buffered saline for 2 h at 4 °C and then incubated overnight with primary antibodies at a 1:1000 dilution in 5% (w/v) Tris-buffered saline containing 0.1% Tween-20. After incubation with secondary antibody for 2 h, proteins were visualized by ECL, and the band intensity was analyzed by using a ChemiDoc XRS densitometer and quantified by Quantity One software (Bio-Rad, Richmond, CA). Protein concentrations were estimated using the BCA method according to the supplier’s recommendations, by using bovine serum albumin as a standard.

### Immunofluorescence

Cells grown on poly d-lysine-coated coverslips were treated with auranofin and/or ANXA5 siRNA in growth medium. The cells were fixed with 4% (w/v) paraformaldehyde in PBS, pH 7.4, for 30 min at 24 °C. After washing with PBS, the cells were blocked for 30 min in PBS containing 5% goat serum and 0.2% Triton X-100, then incubated with primary antibody (1:250) at 4 °C overnight, washed extensively, and stained for 6 h with goat anti-rabbit IgG-Texas Red (1:250). After further washes, the coverslips were mounted on glass slides using Ultra Cruz™ mounting medium. Fluorescence signals were analyzed using an LSM 800 confocal laser scanning microscope (Carl Zeiss, Germany).

### Dual luciferase reporter assay

Cells (1 × 10^6^ cells/well) were cotransfected with 2.5 μg of human COX-2 promoter luciferase constructs and pRL-renilla vectors (Promega, Madison, WI), according to the manufacturer’s protocol, by using the Neon™ transfection system. Luciferase activities were measured consecutively by using the Dual Luciferase Assay System (Promega) with a FilterMax F3 microplate reader (Molecular Devices, CA).

### NF-κB p65 transcription factor assay

Nuclear proteins were extracted from cells by using NE-PER™ Nuclear and Cytoplasmic Extraction Reagents (Thermo Scientific). NF-κB binding activity was measured by using the NF-κB p65 Transcription Factor Assay Kit (Abcam, UK). The assay was carried out as recommended in the assay kit manual.

### PGE2 ELISA assay

Cells were transfected with ANXA5 siRNA and incubated for 48 h in 60-mm plates. The culture supernatants were used for measuring the PGE2 concentration. PGE2 concentration in the culture supernatants were determined by PGE2 high sensitivity ELISA kit (Enzo Life Science).

### Statistical analysis

Statistical analysis was performed using one-way analysis of variance, followed by Dunnett’s pairwise multiple comparison *t*-test, with GraphPad Prism software (GraphPad Software Inc., CA) when appropriate. Differences were considered statistically significant at *P* < 0.05.

## SUPPLEMENTARY MATERIALS FIGURES


