# Supplementary material for: Annexin A5 suppresses cyclooxygenase-2 expression by downregulating the protein kinase C-ζ–nuclear factor-κB signaling pathway in prostate cancer cells
Source: Oncotarget. 2017 Jul 19;8(43):74263–75. doi: 10.18632/oncotarget.19392 (PMC5650338; doi:10.18632/oncotarget.19392)
Supplement: Supplementary file 1 [file oncotarget-08-74263-s001.pdf]

Annexin A5 suppresses cyclooxygenase-2 expression by downregulating the protein kinase C-ζ–nuclear factor-κB signaling pathway in prostate cancer cells

SUPPLEMENTARY MATERIALS

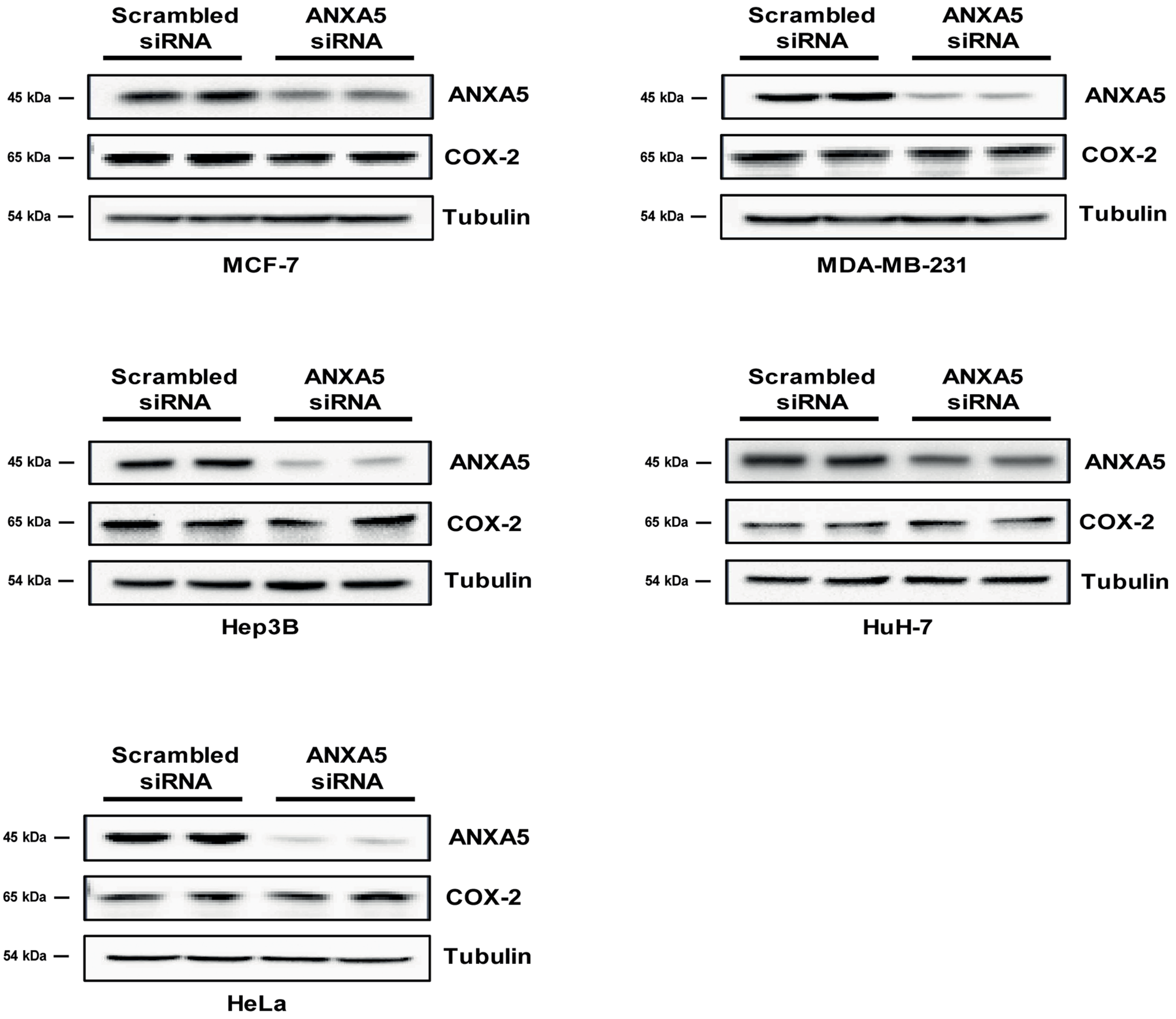

Supplementary Figure 1: Identification of the suppressive-effect on cyclooxygenase 2 (COX-2) expression by inhibition of annexin A5 in cancer cells. Hep3B, HuH-7, HeLa, MCF-7, and MDA-MB-231 cells were transfected with annexin A5 siRNA (37.5 nM) and then western-blot analysis was performed to detect the COX-2 expression by using whole cell lysates.

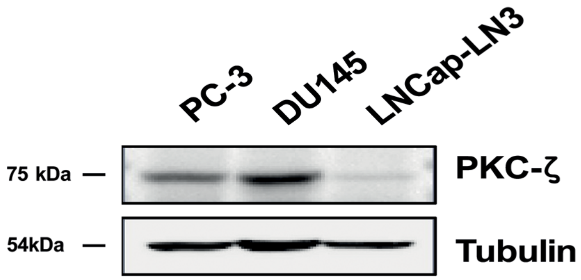

Supplementary Figure 2: Difference of basal level of protein kinase C (PKC)-ζ in prostate cancer cells. Western blot analysis was performed to detect the PKC-ζ basal levels by using the PC-3, LNCaP-LN3, and DU145 whole cell lysates.
